# Serologic evaluation of celiac disease in patients with beta thalassemia major and control 

**Published:** 2015

**Authors:** Iraj Shahramian, Seyed Mohsen Dehghani, Mahmood Haghighat, Noor Mohammad Noori, Ali Reza Teimouri, Elham Sharafi, Manijeh Kalili

**Affiliations:** 1 *Department of Pediatric, Zabol University of Medical Sciences, Zahedan, Iran*; 2 *Department of Pediatric Gastroentrology, Shiraz University of Medical Sciences, Shiraz, Iran*; 3 *Children and Adolescents Health Research Center, Zahedan Medical University, Zahedan, Iran *

**Keywords:** Celiac disease, Beta-thalassemia major, Growth disorder, Disease risk, HLA

## Abstract

**Aim:**

This study reports evaluated prevalence of CD in patients with Beta-thalassemia major.

**Background:**

Celiac Disease (CD) is an autoimmune disorder triggered by ingestion of gluten in genetically predisposed individuals.

**Patients and methods:**

In this case-control study in a period of 3 years, which was performed on 620 children in two groups of Beta-thalassemia major patients (n=200) and control (n=420), serum tissue transglutamianse (tTG) IgA levels were measured. The two groups were compared together in terms of tTG IgA levels, and p<0.05 was considered significant.

**Results:**

The means of serum tTG IgA levels in patients with Beta-thalassemia major and control groups were 28.81±68.44 and 6.94±6.68 U/mL, respectively. There was a significant difference in favor of the case group (p=0.000). Body mass index in the two case and control groups had a significant difference (t=3.859, p=0.001). Belonging to each group will change the probability of having less than 20 in tTG IgA (odds=0.285) and it means that belonging to the control group has a protective role. There is only a significant association in the case of all population (r=0.102, p=0.011). Body mass index in the two case and control groups had a significant difference (t=3.859, p=0.001).

**Conclusion:**

Probability of CD should be considered since the prevalence of CD is high in patients with and Beta-thalassemia major. Patients with thalassemia major are recommended for screening for CD.

## Introduction

 Celiac disease (CD) is an autoimmune mediated gluten sensitive enteropathy that occurs in genetically predisposed individuals (1, 2). There is an increasing incidence of CD due to development of more sensitive serological tests and higher disease consideration (3). The prevalence of CD varies in different subgroups, ranging about 0.5 to 12 percent (4, 5). A higher prevalence of concomitant autoimmune and non-autoimmune diseases, such as thyroid dysfunction and glucose intolerance, has been reported. These associated diseases include portal hypertension, diabetes mellitus, hypothyroidism, Down syndrome, immunoglobulin A deficiency, epilepsy, ulcerative colitis, dermatitis herpetiformis, rheumatoid arthritis, pemphigus, hyperthyroidism, Addison disease, systemic lupus erythematosus, and scleroderma (6). Clinical presentations of CD include malabsorption syndrome encompassed as chronic diarrhea, weight loss and abdominal distention, or atypical non-diarrheal CD. Also, short stature is one of the most common manifestations in both types of CD that is due to GH-Insulin like GH1 axis. In most circumstances, the patients have been presented with atypical form than classic symptoms of malabsorption (7). Beta-thalassemia major is defined as a common genetically hematologic disorder with common endocrine abnormalities including growth hormone (GH) deficiency, diabetes mellitus and hypogonadotropic hypogonadism (8, 9). Short stature is described as a common complication in Beta-thalassemia patients that is caused by GH deficiency (10). Human leukocyte antigen (HLA) system, specially HLADQA1 and DQB1 alleles, represent the major genetic predisposition in CD (11). Likewise, HLADQB1 allele is associated with pathogenesis and susceptibility to Beta-thalassemia major (12-14). Both CD and Beta-thalassemia major are alike in association with autoimmune diseases such as diabetes mellitus, thyroid dysfunction, and glucose intolerance (8,9). In both CD and Beta-thalassemia major, short stature is a well-known feature and this complication in CD could appear even without typical gastrointestinal symptoms. CD bears a close resemblance to Beta-thalassemia major in GH deficiency. Association between CD and Beta-thalassemia major has been established in few case reports. Iron balance is maintained by the control of absorption. Some evidence suggesting that two nutritional deficiency disorders, deficiencies of vitamin A and riboflavin lead to impaired iron absorption or utilization. This appears to account for the contribution that excessive absorption makes to the iron overload seen in patients with iron-loading anemia such as thalassemia major. Atonio Acuaviva study revealed that CD in a young adult male patients with Beta-thalassemia major with an unusual clinical history of hypothyroidism not responding to treatment (15-17). Therefore, this study was conducted to investigate CD in patients with Beta-thalassemia major compared to the control group. 

## Patients and Methods

This case-control study was conducted from 2010 to 2013 on 200 patients with beta-thalassemia major aged 2-16 years old who referred to thalassemia clinics of Zabol and Zahedan University of Medical Sciences, Southeast of Iran, and compared with 420 healthy children in the same age range. Healthy children referred to these centers for annual check-up. All patients underwent physical examination and their medical history was taken. 

Every patient in the case group was diagnosed by complete blood count and hemoglobin electrophoresis and all of them were in regular blood transfusion dependently. In the control group, there was no child with IgA deficiency, or history of digestive, endocrine, and metabolic disorders, iron deficiency, kidney disease, fever, or chronic diseases; they were enrolled after taking the parents’ informed consent.

**Table1:** Nonparametric test in comparison of tTG IgA in case and controls

Variable	Groups	N	Mean Rank	Sum of Ranks	Mann-Whitney U	Wilcoxon W	Z	P value
tTG	case	200	385.505	77101	26999	115409	-7.23274	0.000
	control	420	274.7833	115409				
	Total	620						

**Table 2:** Independent t test in comparison of BMI in case and control groups

Factor	Groups	Mean	S.D	95% Confidence Interval of the Difference		t	P
				LB	UB		
BMI	case	16.9154	2.39524	0.33806	1.03864	3.859	0.001
	control	16.227	1.90612				
	Total	16.449	2.099				

**Table 3:** Independent t test in comparison of BMI and TTG IgA in sex groups

Factor	Groups	Mean	S.D	95% CI		t	P
				LB	UB		
BMI	male	16.7034	2.1466	0.12664	0.79543	2.697	0.007
	female	16.2424	2.03589				
TTG IgA	male	12.84	32.6	-9.545	3.586	-0.691	0.373
	female	15.82	49.01				

**Table 4:** Independent t test in comparison of BMI in tTG IgA status (**< 20 and >=20)**

Factor	Groups	Mean	S.D	95% CI		t	P
				LB	UB		
BMI	tTG Normal	16.41	2.11	-0.89	0.20	-1.24	0.22

Five ml blood was drawn from these children at 8:00 am in fasting. Samples were centrifuged for serum separation and separated serum was held in a -70°C fridge till tTG IgA and total IgA measurements. Finally, they were transferred to the biochemistry laboratory, University of Medical Sciences, Zahedan under the cold chain compliance. Then, 250 microns of the isolated serum of these samples were used for serologic tests with recombinant ELISA. Normal limit of tTG IgA was 20 U/mL. 

 Data analysis was performed using SPSS version 20 and the significance level of 0.05 was considered. 

Quantitative variables were summarized as mean±SD and independent t-test was used for difference. The odds ratio was used for determining the association between tTG IgA status and gender as well groups. 

## Results

A total of 620 children were enrolled and distributed in case and controls as 200 and 420 and grouped in gender as 52.6% (n=326) and 47.4% (n=294) in male and female respectively. The male percentages in case and controls were 71.8% and 28.2% respectively. There was the same trend for females, 63.3% and 36.7%, respectively. Tables 1 shows the results of nonparametric Mann-Whitney U and Wilcoxon won comparing the median of tTG IgA in case and control groups. The non-parametric method was used because of likely bias because of bigger SD than mean for tTG IgA. Mean rank of tTG IgA in case and control were 385.51 and 274.78 respectively. It obviously showed that the rank for case was significantly higher than controls (z=7.23274, p<0.0001).

Tables 2, 3 and 4 show the results of independent t-test in comparison of the two groups. The measures of BMI in two case and control groups had a significant difference in favor of case group, which had a higher value than the control group (t=3.859, p=0.001). Males had higher BMI than the females (t=2.697, p=0.007). Mean of tTG IgA in males and females did not show any differences. Finally, BMI value for normal range (tTG IgA <20) was lower than abnormal range (tTG IgA >=20), but it was not significant (t=-1.235, p=0.217). Table 5 showed sex distribution in tTG IgA according to the cut-off point of 20. In the negative state of tTG (<20), 93% were females while this percentage for males was 95% percent. This distribution did not show any relationship between levels of tTG and sex (r=0.063, p=0.118). The confidence interval for odds ratio (0.383, 1.117) for sex factor revealed that it played the role of both risk and protection. It means that sex had no effect on tTG, but for groups, the 95% CI was equal to (0.095, 0.295) showed that belonging to each group changed the probability of having less than 20 in tTG (odds=0.285) and it means that belonging to the control group had a protective role. Also, the table showed that tTG IgA titers for CD in the case group was 11.5% (23/200), which was significantly higher than the control group with a prevalence of 3.5% (15/420)(p=0.000). Table 6 showed the correlation between BMI and tTG IgA in case and controls and combined population. The table showed that there was only a significant association in combination of population (r=0.102, p=0.011) when there was no relationship between BMI and tTG IgA in case and controls separately. 

**Table 5:** Distribution of tTG IgA gap in sex and groups

Group		tTG		Total	Contingency Coefficient=r	p	odds	95% CI	
		< 20 (Neg)	>=20 (Pos)						
								L	U
Sex	Female	303(93)*	23(7)	326	0.063	0.118	0.654	0.383	1.117
	Male	279(95)	15(5)	294					
	Total	582(94)	38(6)	620					
Groups	Case	177(88.5)	23(11.5)	200	43.434	<0.0001	0.285	0.095	0.297
	Control	405(96.5)	15(3.5)	420					
	Total	582(94)	38(6)	620					

* n (%)

**Table 6:** Correlation of BMI and tTG IgA in terms of case, control and combination

Factor	Statistics	tTG		
		Case and control	Case	Control
BMI	Pearson Correlation	0.102	0.099	0.016
	P value	0.011	0.164	0.739
	Count	620	200	420

## Discussion

The findings of our study showed a significant increase in tTG IgA in patients with Beta-thalassemia major as compared to healthy children and the tTG IgA titers for CD in the case group was 11.5% (23/200) that was significantly higher than the control group with a prevalence of 3.5% (15/420)(p=0.000). According to BMI, there was a significant difference between the two groups and growth failure was observed in the cases versus the controls, showing a significant association. There was a slightly female dominance in total participants (Male/Female=1.1/1). Ankit Parakh reported a 10- year-old boy on a hypertransfusion regimen was referred for early onset growth failure. The case had high tTG IgA level >300 IU/mL (normal value<15) and abnormal jejunal biopsy that confirmed CD diagnosis for patient because the authors concluded the need for CD evaluation even without the classic manifestations in patients with Beta-thalassemia major suffering growth failure (16). As BMI has inverse relationship with height, comparatively we could receive to the result that, in our findings, case groups had higher BMI and consequently the height is lower in which is similar with this case report.

Also, Mangiagli established CD in adolescent thalassemia patients characterized by anorexia, arrest of weight gain and short stature and they emphasized the need to search for CD in all thalassemia patients who present in low stature and arrest of weight gain (17). The finding of this study like Ankit Parakh is just about supporting our result. Clues for CD in these reports were growth failure, hypothyroidism and anorexia that are common in CD and Beta-thalassemia major. The mechanism of growth failure in patients with Beta-thalassemia major, CD nutritional deficiencies, and defects in GH secretion has been proposed as the underlying mechanisms (10, 18). Celiac disease autoimmune basis and association with autoimmune disorders, such as diabetes, thyroid dysfunction, and Addison disease have been demonstrated (6). Additionally, thalassemia patients have autoimmune endocrine abnormalities including GH deficiency, diabetes mellitus, and adrenal insufficiency (10). There is a pro-inflammatory stage, autoimmunity vulnerability, and association with autoimmune diseases (19,20). The other reason for supporting the association between CD and Beta-thalassemia major is genetically similarities in HLA system; specially, HLA DQB1 alleles represent major genetic predisposition in CD and Beta-thalassemia major likewise, pathogenesis and susceptibility to them (11,13,14). According to common findings, such as growth failure, association with endocrine abnormalities, genetic and autoimmune correlation between CD and Beta-thalassemia major is logical. In contrast, Honar conducted an investigation for determining the frequency of CD in children with Beta-thalassemia major with an age under 18 years old versus healthy age /sex matched control group in Shiraz. In their study, there was no association between CD and Beta-thalassemia major and they did not suggest routine screening for CD in children with Beta-thalassemia major (21). We resulted that there was high correlation between CD and Beta-thalassemia major in which is dissimilar with the Honar study. However, in a review article about epidemiology of CD in Iran a very low prevalence of CD in Shiraz province has been reported (22). 

 Makharia reported that from 45 patients with celiac diseases that for 39 subjects serological tests were available in their medical records, 34 from 39 (76%) had positive serological tests for celiac disease. By the most common test for celiac, tTG IgA identified about 23 from 34 (67.6%) when the current study concluded of 11.5% for thalassemia patients (23). 

 In a case report by Ramraj documented that, a 53 years old female with height and weight of 111kg and 161cm (BMI=43.5) respectively and her previous history were overweight at pregnancy, diabetes mellitus (type II), hypertension, thalassemia minor and recurrent diarrhea. This case reported as a patient with celiac diseases after 7 years recurrent diarrhea. One of the main results of our study could be that, people who are underweight and have a short stature have higher risk for celiac diseases, which is in contrast with the Ramraj report (24).

Montuori reported an uncommon CD diagnosis in a 21-year-old thalassemia major female in which after EBV infection, recurrent abdominal pains and diarrhea appeared. She was referred to a gastroenterologist for the reason of persistence of gastrointestinal symptoms. Screening was applied for tTG IgA titter in which was higher than 100 and upper gastrointestinal endoscopy and biopsy confirmed that she had celiac disease (marsh 3a-3b). Celiac as a cause of malabsorption can cause unexpected decrease in ferritin level (25). Ciccocioppo in a case report explained a boy with thalassemia major diagnosed in prenatal and a girl with thalassemia major diagnosed in the ninth month of age. In the case of a girl they observed a reduction in weight and height in childhood. They also understood that the girl had experienced abdominal discomfort and episodes of cramps and diarrhea. The applied some diagnostic process like Montuori and concluded that the girl had celiac disease (26).

In our study 5.5% of patients with thalassemia major had tTG IgA more than 100 in which comparatively is same with two late studies.

Valizadeh reported a misdiagnosed 28-year-old male with thalassemia intermedia who had received frequent blood transfusions and was admitted due to iron deficiency anemia (IDA) and bloody diarrhea. The patient had a history of pemphigus. His physical examination showed pallor, short stature, clubbing and splenomegaly. He had vegetans and chronic diarrhea since childhood. Valizadeh emphasize that diagnosis threshold for celiac disease should be lowered in thalassemia patients who present with short stature. Our results for BMI comparison between case and control groups showed a significant higher level for case group in which these findings are dissimilar (27). 

According to the results of this study the probability of CD should be considered, as the prevalence of CD is high in patients with Beta-thalassemia major. Patients with thalassemia major should be screened for CD, especially cases with associated autoimmune diseases and growth failure even without typical symptoms of CD by measuring tTG IgA and for positive serologic patients performing an intestinal biopsy.
